# The Effect of Cement Addition on Water Vapour Resistance Factor of Rammed Earth

**DOI:** 10.3390/ma14092249

**Published:** 2021-04-27

**Authors:** Piotr Narloch, Wojciech Piątkiewicz, Barbara Pietruszka

**Affiliations:** 1 Faculty of Civil Engineering, Warsaw University of Technology, Al. Armii Ludowej 16, 00-637 Warsaw, Poland; wojciech.piatkiewicz.dokt@pw.edu.pl; 2 Department of Thermal Physics, Acoustics and Environment, Building Research Institute, Ksawerów 21, 00-637 Warsaw, Poland; b.pietruszka@itb.pl

**Keywords:** cement, cement stabilized rammed earth, diffusive resistance, rammed earth, water vapour, water vapour resistance factor, water vapour permeability

## Abstract

The article aims to determine the effect of cement addition on the water vapour resistance factor of stabilized rammed earth. Literature analysis indicates that different earthen materials show large differences in water vapour resistance factor values. The high diffusion resistance of concrete concerning other construction materials suggests that cement will be one of the factors significantly affecting these values. The paper presents water vapour resistance factor test results of rammed earth with various soil particle sizes and cement contents. The obtained results showed that an increase of cement addition increases the diffusion resistance of the material. However, the diffusion resistance of cement stabilized rammed earth is still low compared to concrete.

## 1. Introduction

**Figure 1:**
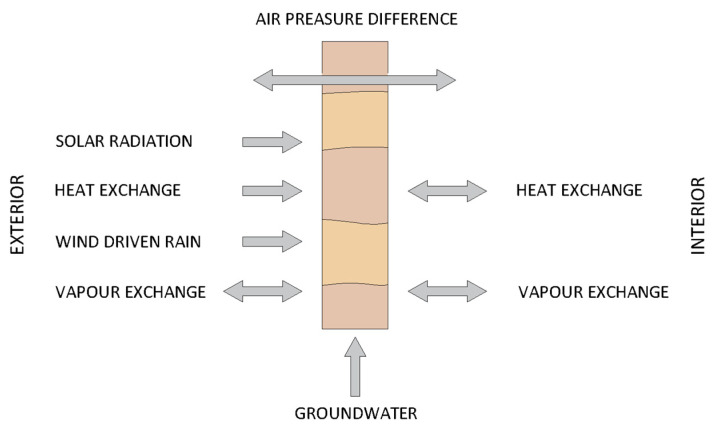
Hydrothermal fluxes and their alternating directions across an earth wall [14].

Rammed earth (RE) is a sustainable building material [1,2,3,4,5,6] whose main component is inorganic soil. Due to its mechanical properties [5,7] and durability [8,9,10], cement is often added to the soil. The erection of cement stabilized rammed earth (CSRE) wall consists of ramming layers of moist soil–cement mixture in formwork. CSRE partitions are usually not plastered [11,12]. However, if they are protected with plaster, they must be properly selected to allow for the evaporation of the moisture contained in the CSRE layer [3,13]. On the exterior surfaces, the walls interact with the outside environment by exchanging heat and water vapour. Similarly, on the interior surfaces, the walls interact with the indoor environment in the same way [11,14] (Figure 1).

Building envelopes separate areas of air with different temperatures and relative humidity, which means that they have different values of partial water vapour pressure. As a result, water vapour diffusion occurs through building envelopes. Building envelopes should be designed in such a way as to avoid water vapour condensation in their interior. Water condensation reduces the durability of building partitions. Moreover, water freezing and thawing in the structure cause the volume of the material to change, inducing the gradual destruction of the material [15]. This leads to the formation of cracks and discoloration in the building partition. Condensation of water vapour in materials also leads to the deterioration of their physical and mechanical properties. Increasing humidity in materials causes an increase in the thermal conductivity coefficient, leading to increased heat losses from the building [15,16,17,18,19]. Such situations are particularly undesirable for energy-efficient buildings, where the heat losses must be reduced as much as possible [16,20] to reach a designed standard. Furthermore, as the humidity increases, the compressive [21,22,23,24] and tensile strength [25,26] of a material decreases. The authors found no study that would link humidity with the water vapour resistance factor. However, it can be inferred that by filling the pores with water, the water vapour resistance factor of rammed earth will rise.

The research [27] investigated CSRE walls in a climatic simulation chamber. The simulator was designed to investigate full-sized building elements with realistic climatic conditions. Four CSRE walls, 300 mm thick, were stabilized with the addition of 6% Portland cement and differed in particle-size distribution. During the experiments, the tested walls were subjected to temperature differences and changing weather conditions, such as relative humidity and rainfall. Despite high humidity levels and temperature differences, sensors mounted inside the walls showed a negligible risk of interstitial and internal condensation.

The article by Brás et al. [28] showed an example of a building whose rammed earth walls were, according to authors, inappropriately designed. The external side of the walls was covered with cement-based plaster with very low permeability, while the internal side of the walls was sealed with paint, glue, insulation material and plasterboard. As a result, the water content in the earthen walls increased nearly to the point of saturation. This induced a drastic drop in the compressive strength of the earthen material, causing the walls to lose stability, and structural failure occurred [28].

Water condensation in building partitions can lead to their destruction as a result of biological and chemical corrosion. The risk of mold growth on damp partitions is also a considerable threat, posing risks to human health (including allergy sufferers, rheumatics) [29,30]. Condensation of water vapour in partitions affects the microclimate of the interior and the esthetics of the partitions [31].

For these reasons, ensuring proper water vapour flow through a building partition is vital so that in given climatic conditions, there is no risk of internal condensation. The heat conduction coefficient and the water vapour resistance factor of the materials from which a partition is constructed have a large impact on the water vapour flow through the partition. The order in which the layers in a building envelope are arranged is also important [15].

This paper presents an analysis of the impact of cement stabilization on the change of the CSRE water vapour resistance factor, μ. This factor is a dimensionless indicator determining the relation between the resistance of diffusion of a material layer and that of an air layer of the same thickness and under the same conditions:

(1) $$\mu =\frac{\delta_{0}}{\delta_{MAT}}$$

where

$\delta_{0}$—water vapour permeability of air;
$\delta_{MAT}$—water vapour permeability of the material;

The water vapour permeability of air depends on the barometric pressure and temperature during the test. It can be calculated by using Formula (2):

(2) $$\delta_{0}=\frac{0.086\cdot p_{0}}{R_{D}\cdot T\cdot p}\cdot {\left(\frac{T}{273}\right)}^{1.81}$$

where

T—thermodynamic temperature (K);
p—barometric pressure (hPa);
$p_{0}$—standard barometric pressure (1013.25 hPa);
$R_{D}$—gas constant of water vapour (462.10^−6^ (Nm/(mg·K)).

The water vapour permeability of a material is defined as the mass of vapour transferred through the sample per second and per unit area and is given by Formula (3):

(3) $$\delta_{\mathrm{MAT}}=W_{p}\cdot d_{\mathrm{MAT}}$$

where

$d_{\mathrm{MAT}}$—mean specimen thickness (m);
$W_{p}$—water vapour permeance (kg/(m^2^·s·Pa)).

The water vapour permeance is defined concerning partial vapour pressure and is given by Formula (4):

(4) $$W_{p}=\frac{G}{A\cdot \Delta_{p_{v}}}$$

where

G—water vapour flow through the specimen (kg/s);
A—area of the specimen (m^2^);
$\Delta_{p_{v}}$—water vapour pressure difference across specimen (Pa).

The area of the specimen (A) is calculated as the arithmetic mean of the free upper and free lower surface areas. The surface area depends on the sealing system used in the test, which may cover part of the sample surface, and therefore, corrections should be made.

Water vapour flow G is the main tested value. It is calculated as the mean of five successive determinations of the change of mass per time Δm_12_. The final value of G is taken as the one in which the last five Δm_12_ are within ±5% of each other [32]. The change of mass per time Δm_12_ is given by Formula (5):

(5) $$\Delta m_{12}=\frac{m_{2}-m_{1}}{t_{2}-t_{1}}$$

where

m_1_ and m_2_—mass of test assembly at time t_1_ and t_2_, respectively (kg);
t_1_ and t_2_—successive times of weighing (s).

**Figure 2:**
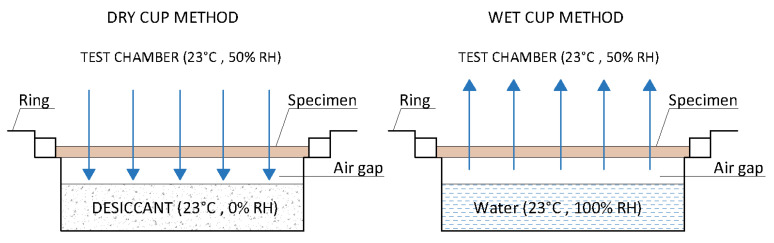
The scheme of dry and wet cup methods.

**Figure 3:**
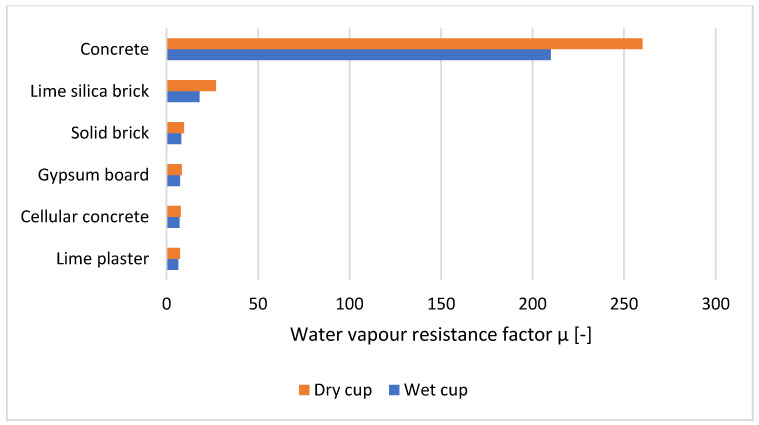
Water vapour resistance factor of building materials [39].

The measurement is made by weighing a sample placed between environments with two different relative humidities until they reach equilibrium. Depending on these relative humidities, the testing method is called “dry cup” or “wet cup” [8,9] (Figure 2). The dry cup method contains a desiccant saturated solution inside the cup, ensuring a humidity of 0–3% [32,33,34]. Calcium chloride [8,9] or silica gel [35] can be used as a desiccant. The wet cup method, on the other hand, contains a saturated aqueous solution, which provides a moisture content with a range of 52–98% [32]. Potassium nitrate KNO_3_ is mostly used as an aqueous solution [36]. Weighings of the specimens should be conducted at a steady state, with the temperature being 23 ± 2 [36]. The dry cup method gives higher values than the wet cup method (Figure 3) [37,38,39].

Earthen materials can assume a wide range of water vapour resistance factors (Table 1). The value of the water vapour resistance factor differs depending on the test direction, which is particularly important in the case of anisotropic earth materials. Maillard and Aubert [34] tested the water vapour resistance factor of unfired clay bricks that have a dry density between 1940 and 2050 kg/m^3^ using the dry cup method. The clay bricks achieved a water vapour resistance factor between 10,6 and 23,1. The test direction of water vapour flow was parallel to the brick forming direction [34]. The samples in which the water vapour flow was perpendicular to the forming direction obtained a value of water vapour resistance factor between 1.31 to 1.9 times higher. For this direction, the water vapour resistance factor achieved values between 19.0 and 44.0 [34]. The flow of water vapour in rammed earth walls takes place perpendicular to the direction in which the ramming occurred [40]. The samples are usually cylindrical in shape and a few centimeters thick [41]. It is easier to prepare them in a direction parallel to the direction of the test. This may indicate that the vapour permeability of some samples made of earthen materials under operating conditions will be higher than the values tested in the cup test [34]. In addition, the study [34] showed the dependence of the water vapour resistance factor on the particle size distribution. It shows that the vapour permeability decreases with the reduction of particle size, e.g., due to the increased proportion of clay in the mixture [17,34,42]. In the study of the vapour permeability of fired clay bricks, the main factor influencing vapour permeability was also the particle size distribution. With the increase of fine clay fractions in the mixture, the vapour permeability decreased [42]. In the CSRE study with a 6% addition of cement, researchers indicate that the main factors affecting the porosity of the material are particle size distribution and the degree of compaction [17]. Increasing the compaction force led to a reduction in porosity between aggregates with a negligible change in porosity inside the aggregate [40]. The water vapour resistance factor of CSRE was measured by Hall and Allinson in [11] using the wet cup method according to EN ISO 12572:2001 [32]. The CSRE mixture contained 7% wt Portland cement. Samples were compacted at their optimum moisture content (OMC) using constant energy of 596 kJ/m^3^ and cured for 28 days at 20 °C and 75% relative humidity. The average value of the water vapour resistance factor for the four test samples was 14.34 [11]. In other tests [37], earth bricks with a similar dry density (1940–2070 kg/m^3^) achieved water vapour resistance factor values between 3 and 7 (wet cup method) and between 7 to 9 (dry cup method). A similarly high vapour permeability was obtained in the study where the rammed earth wall dry density was 1660 kg/m^3^. The water vapour resistance factor for the wall tested by the wet cup method was 4 [12]. In the study [39], rammed earth with a dry density of 1700 kg/m^3^ achieved water vapour resistance factor values ranging from 9.4 to 10.6.

Although the water vapour permeability test appears simple to perform, few results for rammed earth are available (Table 1). The results suggest that earthen materials can be considered permeable materials. A wide literature review indicates the need to study the vapour permeability of compacted soil, especially the influence of stabilizers on this property. Therefore, the paper presents water vapour resistance factor test results of rammed earth with various soil particle sizes and cement contents.

The use of cement stabilizers in rammed earth technology is a common practice today [43,44,45]. The addition of cement increases the mechanical strength and durability of the material [43,44,45,46,47]. On the other hand, it is associated with many disadvantages—including an adverse impact on the environment related to greenhouse gas emissions and pollutants or limiting the possibility of recycling the material [48,49]. Reducing the vapour permeability of stabilized rammed earth is also seen as a disadvantage of using cement [50].

## 2. Materials and Methods

### 2.1. Materials

**Figure 4:**
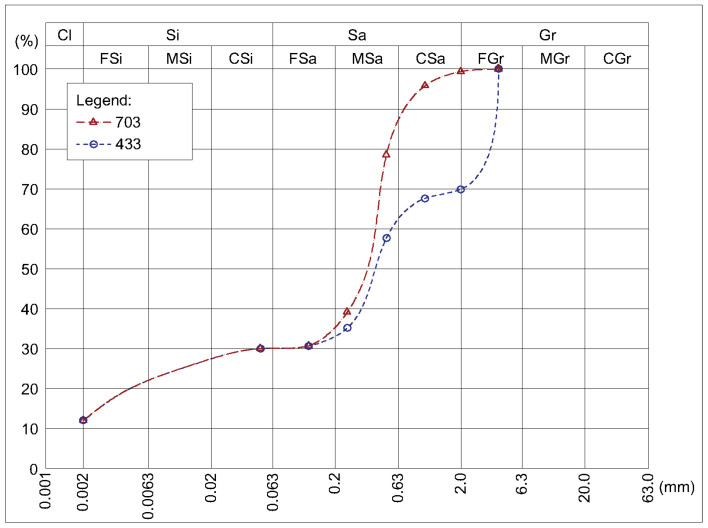
Particle size distribution of soil mixtures used in tests.

The tests were carried out on eight series of samples differing in grain size and cement addition (Table 2). Soil mixtures (Figure 4) were obtained by mixing three components: clay, sand, and gravel, together in a dry state. Clay granularity was determined by aerometric analysis and sand and gravel by sieve analysis. By mixing these ingredients in two different proportions, two soil mixtures were obtained with the particle size distribution shown in Figure 1. One soil mixture (symbol 433) contained 40% sand, 30% gravel, and 30% silt and clay. The other soil mixture (symbol 703) contained 70% sand and 30% silt and clay. To each dry soil mixture, Portland cement CEM I 42.5 R (Odra Cement Plant, Opole, Poland) was added in an amount of 0% to 9% and mixed in a dry state to a homogeneous consistency. Afterward, tap water was added in an amount ensuring optimum moisture content (OMC), i.e., the moisture content at which the maximum dry density was obtained through sample-ramming. OMC depends on both particle size distribution and stabilizer content. For prepared mixtures, depending on the grain size and cement content, the OMC was from 8% to 10% wt (Table 2). Ten samples from each series were prepared for vapour permeability and thermal conductivity tests.

Brito et al. demonstrated that the properties of cement-based materials depend on the geological nature of the aggregates used [51]. The properties of rammed earth depend on the mineral composition of the soil, especially the clay mineral content [52,53,54]. Both soil mixtures contained 30% silty clay. Therefore, their mineral composition was similar. Soils contained about 2.67% swelling minerals (i.e., beidellite—see Table 3).

Cylindrical samples with a diameter of 128 mm and a height of 15 cm were prepared. They were formed in three layers by freely lowering the 6.5 kg manual hand rammer from a height of 30 cm to the surface of the moist soil–cement mixture. Each layer was formed by lowering the manual hand rammer 20 times.

**Figure 5:**
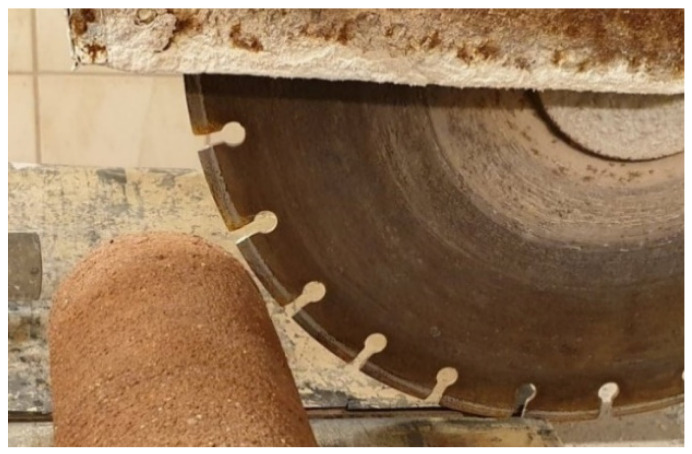
Method of preparing the discs from the cylinder.

**Figure 6:**
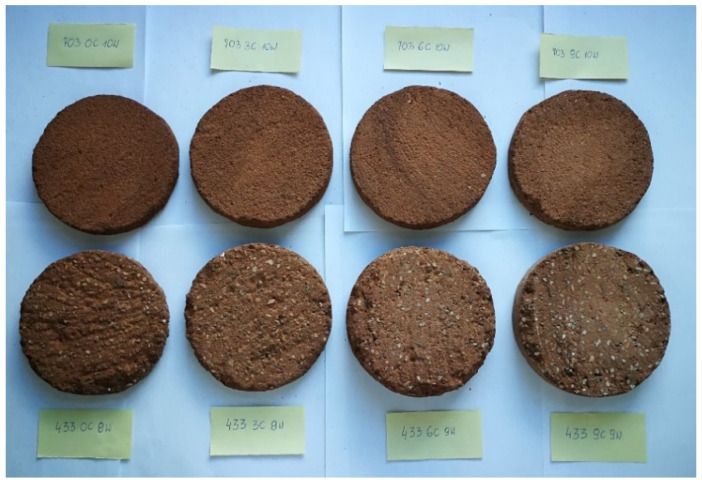
Samples for diffusion resistance coefficient test.

The resulting cylindrical samples were cured for 28 days at a temperature of 23 ± 5 °C and relative humidity of 50 ± 5%. Discs 3 cm high were cut from the center of the cylindrical samples with a table saw (Figure 5). Samples prepared in this way (Figure 6) were first subjected to the water vapour resistance factor test. Next, discs with a diameter of 5 cm and a height of 22 mm were cut out from these samples and subjected to the thermal conductivity test.

### 2.2. Methods

#### 2.2.1. Vapour Permeability Test

**Figure 7:**
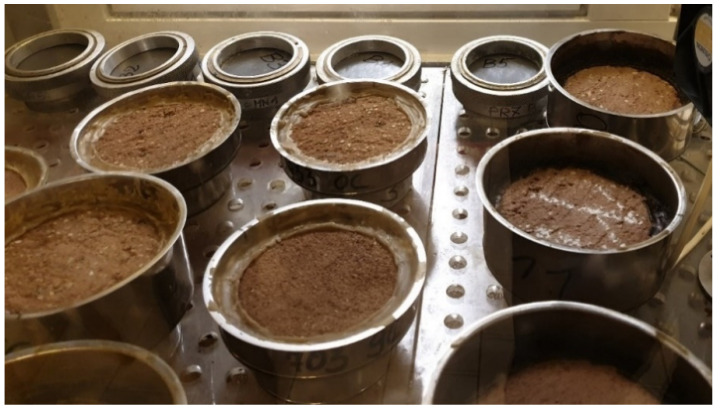
Vapour permeability test.

The tests were performed using the dry cup method according to ISO 12572:2016 [32]. Each test series consisted of 10 samples. Before testing, the samples were first dried at 40 °C for 24 h, then at 60 °C and 80 °C after 48 h. Next, the samples were conditioned at 23 ± 3 °C and 50 ± 3% relative humidity until a constant weight was obtained. Subsequently, at the bottom of each vessel, a layer of moisture absorber was placed—calcium chloride (CaCl_2_). The distance between the sample and the moisture absorber was 15 mm. Using melted wax with the addition of a plasticizer. The tested samples were tightly attached to the open side of the vessel (Figure 7).

Every 24 h, the weight changes of the samples were measured. When five consecutive changes in mass per unit of time were constant (i.e., within ±5% of the mean for each sample), the study was terminated. At each weighing, the conditions prevailing in the test chamber were recorded, and it was checked whether they were within the standard [33].

#### 2.2.2. Thermal Conductivity Test

An additional test carried out as part of the study was the thermal conductivity test. The test was conducted following standard EN 12664 [55]. Samples for this test were obtained by cutting smaller samples from those prepared for the diffusion resistance coefficient test. The samples were cylindrical with a diameter of 50 mm and a height of 22 mm. Before testing, the samples were conditioned at 23 ± 3 °C and 50 ± 3% humidity until a constant weight was obtained.

**Figure 8:**
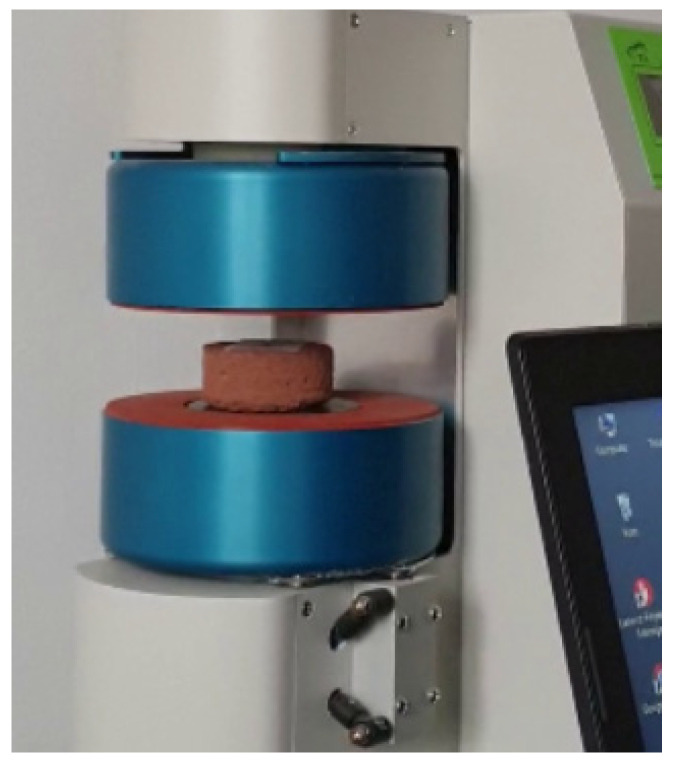
Thermal conductivity test of one of the CSRE samples in FOX 50 plate apparatus.

Determination of the heat transfer coefficient under steady heat flow conditions was performed using a single-sample FOX 50 plate apparatus with heat flux density sensors in a horizontal orientation (Figure 8). The device has a measuring range of 0.1 to 10 W/mK. Measurements were made at an average sample temperature of 10 °C, a temperature difference over a sample thickness of 10 K, and heat transfer from the bottom up at an ambient temperature of 22.4 °C.

## 3. Results

**Figure 9:**
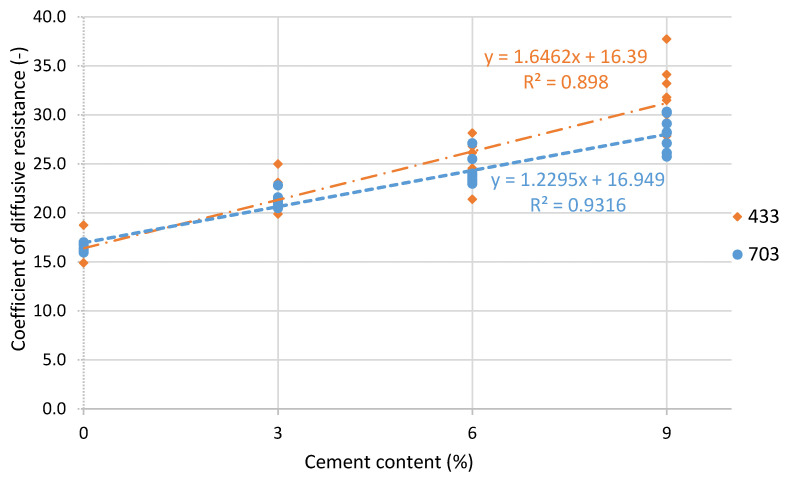
Effect of cement addition of CSRE coefficient of diffusive resistance (linear regression).

The results of the tests are summarized in Table 4. The results indicate an almost linear relationship between the addition of cement and the value of the water vapour resistance factor for both tested soil mixtures (Figure 9). Likewise, the coefficients of the determination indicate that for both series of samples, linear models are a good fit. The determination coefficients are 0.93 for mixture 703 and 0.90 for mixture 433. With the addition of 9% cement, a significant increase in the water vapour resistance factor was observed (by 65% and 82% for mixture 703 and 433, respectively).

**Figure 10:**
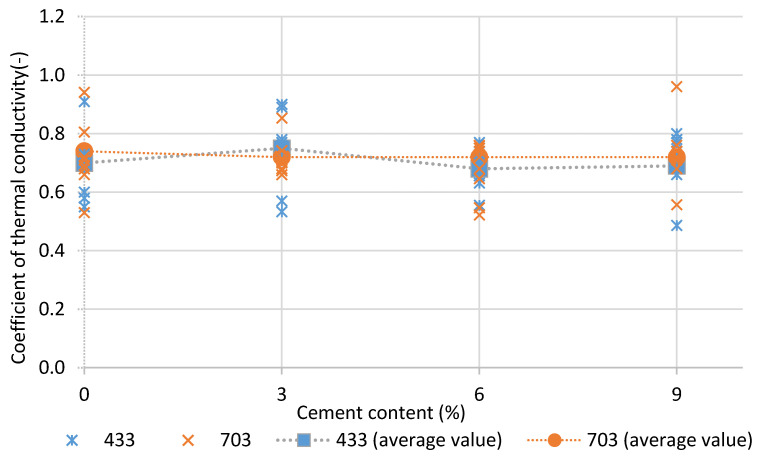
Results of CSRE coefficient of thermal conductivity.

There was no change in the average coefficient of thermal conductivity due to the addition of cement (Figure 10). The determination coefficients for linear regression were close to 0, which resulted from the small size of the tested samples. The field of measurement in the FOX 50 plate apparatus is a circle with a diameter of 2 cm. The nonhomogeneous structure resulting from the granularity and air gaps in the sample significantly affect the local thermal conductivity result. The average dry density results showed that the addition of cement increased the dry density of the material.

## 4. Discussion

The obtained CSRE water vapour resistance factor results determined by the same measuring method (dry cup) are higher than the value given in the literature (Table 1). This may be due to the different particle size distribution of the tested samples. In this study, the authors used a mixture that contained a 12% clay fraction (see Figure 4). Table 1 consists mostly of earthen materials, such as extruded earth bricks, unfired clay bricks, rammed earth, or SRE, containing a higher clay fraction [12,34,37,39]. Many studies have highlighted the significant influence of particle size distributions on the vapour permeability of rammed earth [17,34,36,42].

The authors of this study showed an almost linear relationship between the addition of cement and the value of the water vapour resistance factor. The addition of cement changes the particle size distribution by increasing the proportion of very fine particles in the mixture. Fine cement particles fill the pores of rammed earth, and the material is sealed.

Despite the low clay fraction content compared to those reported in the literature, the water vapour resistance factor of the tested samples without cement addition was relatively high. The water vapour flow in the test was parallel to the forming direction, which means that the value of the water vapour resistance factor in the perpendicular direction will be even greater [34]. The differences in vapour permeability compared to the literature also result from the difference in the density of the samples, which is affected by the composition of the mixture and the force of compaction, as suggested in [40].

The use of cement additive ensures rammed earth durability in a cool climate (9% according to [56]), increases the diffusion resistance of the material, and practically does not affect its thermal conductivity. This is important information that is needed to properly design an external partition in a cold climate, in which the external partition requires thermal insulation. The value of the water vapour resistance factor of unstabilized rammed earth and rammed earth stabilized with 9% cement is almost doubled. Therefore, the amount of cement addition may affect the design of the external partition in a cool climate.

Rammed earth can also be stabilized with binders other than cement. Another popular stabilizer is lime, which also improves the material’s resistance to water [39,43,57]. Typically lime is added in the amount of 6% to 12% by weight [39]. As shown in Table 1, rammed earth stabilized with 6% lime addition is characterized by a diffusion resistance coefficient of approx. 9.4–10.6, thus lower than all the values obtained in the tests. This is most likely because, in the research [39], the samples had a dry density of 1700 kg/m^3^ and a different grain size. Therefore, it is not possible to compare the influence of cement and lime stabilization on the rammed earth diffusion resistance based on the results provided.

It is possible to modify earthen materials with other substances, such as silicon nanoparticles, titania and silica nanoparticles, silane–siloxane, beeswax and NaOH solutions [12,40]. These modifications led to a reduction in water absorption, the moisture buffering capacity MBV (4.2 to 1.4) [40], and increasing the water vapour resistance factor (from 8 to 10) [12]. There is a way to stabilize earthen materials with microbes. Microbes can have a positive effect on the properties of rammed earth. In the study [24], CSRE blocks with 6% cement addition were used. Additionally, the blocks were cured with ureolytic bacteria. The stabilization of the CSRE with microbes led to a reduction in water absorption, which translates into increased strength and durability [24]. This shows that further research is needed to assess the influence of other stabilizers as well as methods of curing RE samples on their water vapour diffusion resistance.

## 5. Conclusions

In the research presented by this paper, unstabilized rammed earth composed of different soil mixtures obtained a water vapour resistance factor of 16.6. With the addition of 9% cement, water vapour resistance factors between 27.9 and 31.8 were obtained depending on the soil particle size. An almost linear relationship between the addition of cement and the value of the water vapour resistance factor was observed. The addition of cement did not significantly change the coefficient of thermal conductivity. In this study, the authors obtained higher values of the water vapour resistance factor than in the literature. This may be due to higher density and different particle size distributions. Along with an increase in the proportion of cement in the mixture, the proportion of fine fractions increased, and the diffusion resistance fell with it. Despite the increase in the water vapour resistance factor when using cement, the material still has low diffusion resistance compared to concrete. In this study, the authors obtained higher values of the water vapour resistance factor than in the studied literature (Table 1), which could have been caused by a different particle size distribution and a higher content of very fine fractions in the mixture. The addition of cement may affect the risk of water vapour condensation in building envelopes using rammed earth construction layers. There are other stabilizers for rammed earth. Therefore, further research should assess the influence of their addition on changes in the diffusion resistance of rammed earth.

## Figures and Tables

**Table 1:** Water vapour resistance factor of earthen materials.

Material	Stabilizer	Clay Fraction (<0.002 mm)	Density (kg/m^3^)	Method	Water Vapour Resistance Factor μ (-)	Reference
RE	0%	39%	1660	Wet	4	[12]
Unfired clay bricks	0%	31–58%	1940–2050	Dry	10.6–23.1	[34]
					19.0–44.0	
Extruded earth bricks	0%	23–38%	1940–2070	Wet	3–7	[37]
				Dry	7–9	
SRE	6% lime	16%	1700	Dry	9.4–10.6	[39]
CSRE	7% cement	-	1900	Wet	14.34	[11]

**Table 2:** Sample series used in the tests.

Sample Series	Soil Mixture	Cement Addition (%)	Water Content (%) (Equal to OMC)
703 C 0%	703	0	10
703 C 3%	703	3	10
703 C 6%	703	6	10
703 C 9%	703	9	10
433 C 0%	433	0	8
433 C 3%	433	3	8
433 C 6%	433	6	9
433 C 9%	433	9	9

**Table 3:** Mineral composition of soil mixtures used in tests.

Mineral Composition (%)									
Mixtures	Clay Minerals	Including:			Goethite	Siderite	Carbonates	Organic Substance	Quartz and Carbonate Crumbs
		Beidellite	Kaolinite	Illite					
703	13.11	2.67	2.58	7.86	-	1.8	-	-	85.09
433	13.11	2.67	2.58	7.86	-	1.8	-	-	85.09

**Table 4:** Comparison of the results of heat conduction and diffusion resistance for the tested CSRE series.

Sample Series	Average Dry Density (kg/m^3^)	Average Water Vapour Resistance Factor (-)	Average Coefficient of Thermal Conductivity (W/(mK))
703 C 0%	2093	16.6	0.74
703 C 3%	2085	21.3	0.72
703 C 6%	2106	24.1	0.72
703 C 9%	2186	27.9	0.72
433 C 0%	2158	16.6	0.70
433 C 3%	2155	21.5	0.75
433 C 6%	2167	25.4	0.68
433 C 9%	2187	31.8	0.69

## Data Availability

The data presented in this study are available on request from the corresponding author.
